# Characterization of the complete mitogenome of *Anopheles aquasalis*, and phylogenetic divergences among *Anopheles* from diverse geographic zones

**DOI:** 10.1371/journal.pone.0219523

**Published:** 2019-09-03

**Authors:** Luis Martinez-Villegas, Juliana Assis-Geraldo, Leonardo B. Koerich, Travis C. Collier, Yoosook Lee, Bradley J. Main, Nilton B. Rodrigues, Alessandra S. Orfano, Ana C. A. M. Pires, Thais B. Campolina, Rafael Nacif-Pimenta, Djane C. Baia-da-Silva, Ana P. M. Duarte, Ana C. Bahia, Claudia M. Rios-Velásquez, Marcus V. G. Lacerda, Wuelton M. Monteiro, Gregory C. Lanzaro, Nagila F. C. Secundino, Paulo F. P. Pimenta

**Affiliations:** 1 Laboratory of Medical Entomology, Institute René Rachou, Oswaldo Cruz Foundation, Minas Gerais, FIOCRUZ, Belo Horizonte, MG, Brazil; 2 Biosystems Informatics and Genomics Group, Institute René Rachou, Oswaldo Cruz Foundation, Minas Gerais, FIOCRUZ, Belo Horizonte, MG, Brazil; 3 Laboratory of Physiology of Haematophagous Insects, Federal University of Minas Gerais, Belo Horizonte, MG, Brazil; 4 Daniel K. Inouye US Pacific Basin Agricultural Research Center (PBARC), United States Department of Agriculture, Agricultural Research Service, Hilo, Hawaii, United States of America; 5 Vector Genetics Laboratory, Department of Pathology, Microbiology and Immunology, School of Veterinary Medicine, University of California-Davis, Davis, California, United States of America; 6 Davis Arbovirus Research and Training, School of Veterinary Medicine, University of California-Davis, Davis, California, United States of America; 7 Institute of Clinical Research Borborema, Tropical Medicine Foundation Dr. Heitor Vieira Dourado, Manaus, AM, Brazil; 8 Graduation Program in Tropical Medicine, Amazonas State University, Manaus, AM, Brazil; 9 Foundation of Tropical Medicine Dr. Heitor Vieira Dourado, Manaus, AM, Brazil; 10 Institute of Biophysics Carlos Chagas Filho, Federal University of Rio de Janeiro, Rio de Janeiro, Brazil; 11 Institute Leonidas and Maria Deane, Oswaldo Cruz Foundation, FIOCRUZ, Manaus, AM, Brazil; Virginia Polytechnic Institute and State University, UNITED STATES

## Abstract

Whole mitogenome sequences (mtDNA) have been exploited for insect ecology studies, using them as molecular markers to reconstruct phylogenies, or to infer phylogeographic relationships and gene flow. Recent *Anopheles* phylogenomic studies have provided information regarding the time of deep lineage divergences within the genus. Here we report the complete 15,393 bp mtDNA sequences of *Anopheles aquasalis*, a Neotropical human malaria vector. When comparing its structure and base composition with other relevant and available anopheline mitogenomes, high similarity and conserved genomic features were observed. Furthermore, 22 mtDNA sequences comprising anopheline and Dipteran sibling species were analyzed to reconstruct phylogenies and estimate dates of divergence between taxa. Phylogenetic analysis using complete mtDNA sequences suggests that *A*. *aquasalis* diverged from the *Anopheles albitarsis* complex ~28 million years ago (MYA), and ~38 MYA from *Anopheles darlingi*. Bayesian analysis suggests that the most recent ancestor of *Nyssorhynchus* and *Anopheles* + *Cellia* was extant ~83 MYA, corroborating current estimates of ~79–100 MYA. Additional sampling and publication of African, Asian, and North American anopheline mitogenomes would improve the resolution of the *Anopheles* phylogeny and clarify early continental dispersal routes.

## Introduction

The mitogenome of most insects is composed of a small double-stranded circular molecule of 14–20 kb in length. It contains 37 genes including 13 protein-coding genes (PCG), 22 transfer RNA genes (tRNA) and two ribosomal RNA genes (small (srRNA) and large (lr-RNA) ribosomal subunits). Additionally, it contains an A+T rich control region that is involved in the initiation of transcription and replication [1, 2]. The order of the genes within the mitogenome is highly conserved and can be traced to the ancestral gene arrangement from the Bilateria [3], which differs slightly from ancestral ecdysozoan and arthropod mitogenomes [4].

A total of 475 formally recognized species of *Anopheles* are currently known [5]. Until recently, knowledge regarding the evolution, divergence time, and phylogenetic relationships among representative species within this genus were scarce. Despite its medical importance worldwide, this gap has been due to the relevance and focus on African anophelines [6–9]. The widespread existence of cryptic species complicates taxonomic and phylogenetic analyses in the genus *Anopheles* [6, 7, 10, 11]. Nevertheless, the recent publication of 16 anopheline genomes [8] may provide, in the near future, new full mitogenomes as parallel assemblies of the genomic data produced. This would enable more accurate phylogenomic reconstructions and enhance estimates of the divergence times among members of this genus.

The current hypothesis regarding *Anopheles* evolution is mostly based on the geographic distribution of extant species [12]. It proposes that major mosquito lineages, including *Anopheles*, originated in Western Gondwana approximately 145 to 100 million years ago (MYA) during the periods recognized as late Jurassic or early Cretaceous [12, 13]. The genus *Anopheles* would have emerged in what now is South America and, following rapid diversification and migration across land bridges, colonized most of the Earth’s favorable habitats [12, 14]. Recent publications hypothesize that the *Anopheles* subgenera *Nyssorhynchus* and *Anopheles* + *Cellia* diverged between 79–100 MYA [6, 15, 16], suggesting that their most recent common ancestor might have lived before the geological split of Western Gondwana ~95 MYA [16].

The human malaria cycle probably evolved in Africa, where first interactions between parasites, their anopheline hosts, and hominids occurred [7]. As malaria-infected humans migrated out of Africa, they carried *Plasmodium* parasites with them, leaving their African mosquito vectors behind. This migration gave rise to a journey of adaptation between the *Plasmodium* parasite and no less than 34 different anopheline mosquito species worldwide [17]. Based on available evidence of historical, archaeological and genetic type, it is believed that human malaria was introduced into the Americas by Europeans who transferred both, *Plasmodium falciparum* and *Plasmodium vivax* (the most prevalent malaria parasite species) to the indigenous population [18–20]. Although Neotropical anophelines were already adapted to feed upon primates, including humans, interactions between Neotropical malaria vectors, humans, and malaria parasites, can be considered geologically and evolutionarily recent [7, 16].

*Anopheles aquasalis* is a relevant Neotropical malaria vector of *P*. *vivax* on the Atlantic and Pacific coasts from Central America to Southern Brazil. *An*. *aquasalis* has been reared under laboratory conditions since 1995, being a well-established model for experimental studies involving the interaction of malaria vectors with several *Plasmodium* species [21]. Nonetheless, little is known about its evolutionary relationship with other *Anopheles* sibling species.

Mitochondrial genome sequence data are useful to infer phylogenetic and phylogeographic relationships [22–26]. Here we present a preliminary characterization of the mitogenome of *A*. *aquasalis* assembled by Next Generation shotgun sequencing (NGS). We compared the mtDNA genome sequence, and some of its features, with other selected anopheline mitogenomes. We applied Bayesian analysis to reconstruct phylogenetic relationships and estimate divergence times amongst other human malaria vectors. The implications of these findings were briefly discussed regarding the evolutionary history of anophelines in general. A more thorough and balanced analysis of anopheline mitogenomes, including representatives from North America, Asia and Africa would provide an in-depth description of dispersal routes throughout evolutionary and geological times.

## Methods

### *Anopheles aquasalis* colony

*A*. *aquasalis* were obtained from a colony established at the Medical Entomology Laboratory at Instituto de Pesquisas René Rachou-FIOCRUZ (Fiocruz, Minas Gerais). The mosquitoes originally came from a colony established in 1995 in Rio de Janeiro [27, 28], and are currently kept under laboratory conditions as previously described [29].

### Single mosquito DNA extraction

Genomic DNA from a single adult female *A*. *aquasalis* was extracted using the QiagenDNeasy blood and tissue kit (Qiagen, Hilden, Germany) according to the protocol for purification of insect DNA with a minor modification: Qiagen EB buffer rather than AE buffer was used to avoid possible interference of EDTA with the Nextera kit enzymes. The purified *Anopheles* genomic DNA was then quantified with the Qubit HS (Life Technologies, USA) system and used to construct the genomic library.

### Whole genome shotgun sequencing

The genomic DNA was processed using the Nextera DNA sample preparation kit (Epicentre Biotechnologies, Madison, WI). Thirty nanograms of sample DNA were fragmented utilizing 5 μl of Tagment DNA Enzyme with 25 μl of Tagment Buffer. Tagmentation reactions included in the Nextera kit were performed by incubating the sample for 5 min at 55°C. The tagmented DNA was purified using the QiagenMinElute protocol (QIAGEN, Germany). Purified DNA was eluted from the column with 11 μl of nuclease-free water. Purified DNA (5 μl) was used as the template in a 20-μl volume for limited-cycle PCR (5 cycles) and processed as outlined in the Nextera protocol (Illumina). Amplified DNA was purified using the AMPure Bead cleanup (Beckman Coulter, USA) according to the manufacturer's protocol. The fragment size distribution of the tagmented DNA was analyzed utilizing a 2100 Bioanalyzer with a 7500 DNA assay kit (Agilent Technologies, Santa Clara, CA). Fragments of ~600 bp long were carried out for sequencing. The library was sequenced on one lane of an Illumina HiSeq2000 instrument to generate paired-end reads. Sequencing was performed by The Vincent J. Coates Genomics Sequencing Laboratory (GSL) at the University of California, Berkeley.

### Mitochondrial genome assembly

Sequences were assembled *de novo* using Velvet v1.2.10 [30] with a k-mer size of 41, according to the scripts and parameters suggested by the Velvet Manual (https://www.ebi.ac.uk/~zerbino/velvet/Manual.pdf) and in-house protocols from the UC Davis Vector Genetics Laboratory. The assembled contigs were aligned to the mtDNA sequence of *An*. *gambiae* (GenBank No. L20934.1) and *A*. *darlingi* (GenBank No.GQ918273.1) using the MUMmerv3.0 software [31] to identify and confirm the *de novo* assembly of the *A*. *aquasalis* mitogenome.

### Sequence analysis: Composition and genomic features of the *A*. *aquasalis* mitogenome

The assembled mitogenome was manually inspected for repeats at the beginning and end of the assembly to infer circularity. Automatic annotation of the mitogenome fasta file was performed with MITOS [32], followed by a manual curation based on the GenBank file format. Manual inspection of the predicted Protein-Coding Genes (PCG's), ribosomal RNA (rRNA) genes, transfer RNA (tRNA) genes, and the AT rich region was performed with Artemis -release 16- [33]. The nucleotide sequences of PCGs were translated based on the invertebrate mtDNA genetic code.

The manual curation of coding regions and rRNA genes was mainly carried by sequence comparison with published insect mitogenome sequences such as *A*. *gambiae* (GenBank No. L20934.1) and *A*. *darlingi* (GenBank No.GQ918273.1) amongst others. Careful attention was given to PCGs comparing the predicted Open reading frames (ORFs) to the UniProt database (http://www.uniprot.org/) giving more weight to similarity hits that had experimental validation.

The MITOS annotated tRNAs were verified and their secondary structures predicted with the tRNAscan-SE search server v1.21 [34] with default settings: the invertebrate mitochondrial codon predictors, and a cove score cut off of 5 (software tool available at: http://lowelab.ucsc.edu/tRNAscan-SE/). Some tRNA genes could not be detected by tRNAscan-SE. They were identified by direct comparison and sequence similarity to tRNAs of other dipterans or anophelines. These tRNA genes were modeled with RNAStructure [35]. To maintain a uniform format, all of the 22 figures were generated with RNAStructure.

To visualize the annotated mitogenome, circular representation of it was generated with Blast Ring Image Generator (BRIG) [36]. For such purpose, a GenBank formatted file and the fasta file were employed according to the Brig 0.95 Manual. Available at: http://ufpr.dl.sourceforge.net/project/brig/BRIGMANUAL.pdf.

Nucleotide composition analyses, expressed as AT%, were performed for individual PCGs, full mtDNA, concatenated PCGs, concatenated tRNAs, lrRNA (*16S*), srRNA (*12S*) and concatenated rRNAs. For the aforementioned targets, composition bias based on strand asymmetry values were estimated using the following formulae for skews: AT skew = [A-T]/[A+T] and GC skew = [G-C]/[G+C] as proposed by Perna & Kocher (1995) [37] on an Excel spreadsheet. Codon bias was assessed estimating the relative synonymous codon usage (RSCU). All the above compositional analyses (except bias estimation) were performed using MEGA v6.0 [38]. The phylogenetic and comparative analyses performed henceforth relied upon available sequences and published literature regarding anopheline and culicine species detailed in Table 1.

**Table 1:** List of the insect species used in this study with their corresponding GenBank number.

Species	Family	Length (bp)	GenBank No.	Vector of malaria in (continent)	Reference
*Anopheles aquasalis*	*Culicidae*	15393	NJHH00000000	South America	This study
*Anopheles punctulatus* (isolate ITN_PNG-18)	*Culicidae*	15412	JX219738.1	Oceania	[6]
*Anopheles farauti* 4 (isolate 7_10–11)	*Culicidae*	15412	JX219735.1	Oceania	[6]
*Anopheles farauti* 4 *(isolate 8_11–12)*	*Culicidae*	15412	JX219736.1	Oceania	[6]
*Anopheles hinesorum*	*Culicidae*	15336	JX219734.1	Oceania	[6]
*Anopheles koliensis* (isolate ESP001B)	*Culicidae*	15412	JX219743.1	Oceania	[6]
*Anopheles dirus* A (isolate A1)	*Culicidae*	15404	JX219731.1	Southeast Asia	[6]
*Anopheles dirus* A (isolate A2)	*Culicidae*	15404	JX219732.1	Southeast Asia	[6]
*Anopheles cracens* (isolate B1)	*Culicidae*	15412	JX219733.1	Southeast Asia	[6]
*Anopheles albitarsis* F	*Culicidae*	15418	HQ335349.1	South America	[39]
*Anopheles albitarsis* G	*Culicidae*	15474	HQ335346.1	South America	[39]
*Anopheles deaneorum*	*Culicidae*	15424	HQ335347.1	South America	[39]
*Anopheles janconnae*	*Culicidae*	15425	HQ335348.1	South America	[39]
*Anopheles oryzalimnetes*	*Culicidae*	15422	HQ335345.1	South America	[39]
*Anopheles darlingi North*	*Culicidae*	15386	GQ918272.1	Central America	[40]
*Anopheles darlingi South*	*Culicidae*	15385	GQ918273.1	South America	[40]
*Anopheles quadrimaculatus* (A strain Orlando)	*Culicidae*	15455	L04272.1	North America	[41]
*Anopheles gambiae*	*Culicidae*	15363	L20934.1	Africa	[42]
*Culex pipiens*	*Culicidae*	14856	HQ724614.1	NA	[43]
*Aedes aegypti*	*Culicidae*	16655	EU352212.1	NA	Unpublished
*Aedes albopictus*	*Culicidae*	16665	NC_006817.1	NA	Unpublished
*Drosophila melanogaster*	*Drosophillidae*	19517	U37541.1	NA	[44]

For anopheline species, the continent in which they exert their malaria vectorial activity is shown. The sequence length reflects the number of base pairs assembled not considering Ns.

### Comparative analyses between anophelines from different geographic regions

Comparative analyses regarding nucleotide composition and strand asymmetry were performed between *A*. *aquasalis* and four other anophelines representative of different geographic regions: *Anopheles punctulatus* (GenBank No. JX219738.1) from South East Asia, *A*. *gambiae* (GenBank No. L20934.1) from Africa, *A*. *darlingi* North (GenBank No. GQ918272.1) from Central America, and *A*. *darlingi* South (GenBank No. GQ918273.1) from South America. For each mtDNA genome, base composition (expressed as AT%) and strand asymmetry (AT and GC skew) were calculated as explained above.

Additionally, a CDS nucleotide similarity comparison between *A*. *aquasalis*, the above-cited anophelines, plus *Anopheles albitarsis* (GenBank No. HQ335349.1)—another South American brackish-water tolerant species—was performed using the Blast2sequence online tool [45] available at http://blast.ncbi.nlm.nih.gov/Blast.cgi#.

### Phylogenetic analysis and molecular dating

To further our knowledge on the *Anopheles* genus phylogeny, as well as to estimate the divergence time or split between *A*. *aquasalis* and other Neotropical anophelines, we reproduced the phylogeny and molecular dating analyses performed by Logue *et al*., (2013) [6] using the tools and parameters suggested by the authors adding the assembled *A*. *aquasalis* mitogenome.

Briefly, 21 insect mitogenomes were selected from the ones used by Logue *et al*., (2013) [6] and their sequences retrieved from the NCBI databank (Table 1). Then, for each of the 13 PCGs, the following actions were performed: the DNA sequences were translated into amino acid sequences using the invertebrate mt genetic code, then they were aligned to each other with the MAFFT alignment engine, and the aligned amino acid sequences were reverse-translated back into nucleotide sequences. All the above steps were performed with the online tool Translator X [46] using default parameters (available at: http://translatorx.co.uk/). Afterward, the aligned sequences from all 13 mt genes were concatenated using FASconCAT [47]. The concatenated PCG sequences from the 22 mitogenomes were analyzed with jModeltest v0.1.1 [48] to determine the best nucleotide substitution model according to the Akaike Information Criterion.

Bayesian phylogenies were reconstructed, and node ages inferred, using BEAST v1.7.5 [49]. The following program parameters were used: an uncorrelated lognormal relaxed clock model allowing for rate heterogeneity among species; the GTR + G substitution model; the SRD06 model of partitioning, which enables estimation of nucleotide substitution parameters separately for the 1^st^ + 2^nd^ and 3^rd^ codon positions (this apparently provides a better fit for protein-coding nucleotide data), and a Yule model for tree reconstruction. With the above parameters, three independent runs of 20 million generations were performed, saving the generated trees every 1,000 generations. All runs were then combined after a burn-in of 10% using LogCombiner v1.7.2 and afterwards, Tracer v1.5 was used to verify the mixing of the Markov chains (both tools are part of the BEAST pipeline). The maximum credibility tree was determined using TreeAnnotator v1.7.2 and visualized with FigTree v1.4.3 available at http://tree.bio.ed.ac.uk/software/figtree/. Divergence times were estimated using BEAST v1.7.5 following the instructions provided by the developers at http://beast.bio.ed.ac.uk/. In addition to the aforementioned parameters, the *Drosophila*-*Anopheles* divergence time was set as the calibration point using a prior distribution normally distributed around a mean of 260 million years ago (MYA) ranging from 243 to 276 MYA as suggested by Gaunt & Miles (2002) [50].

## Results

### Composition and genomic features

**Fig 1:**
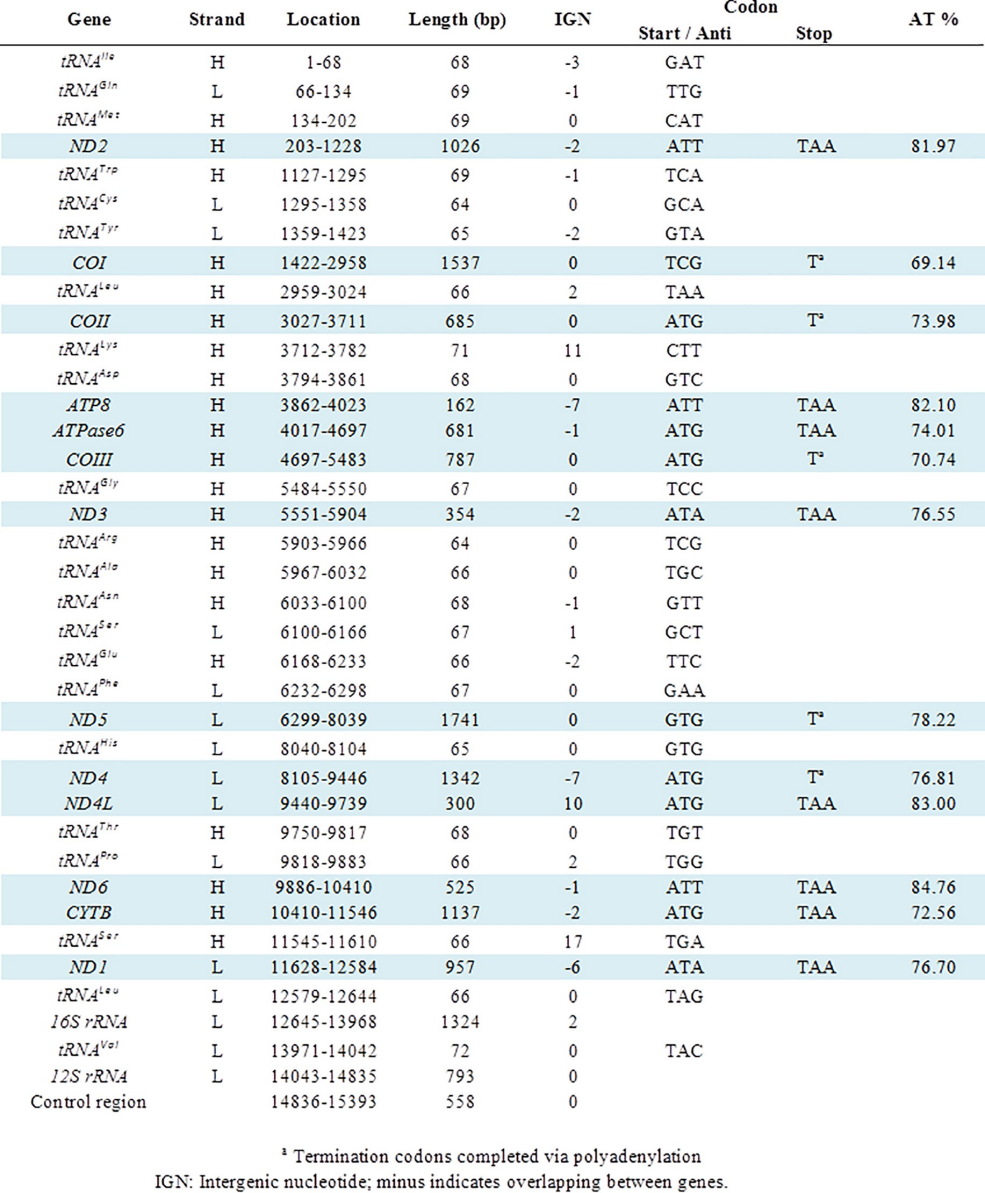
Organization and gene features of the *A*. *aquasalis* mitochondrial genome.

**Fig 2:**
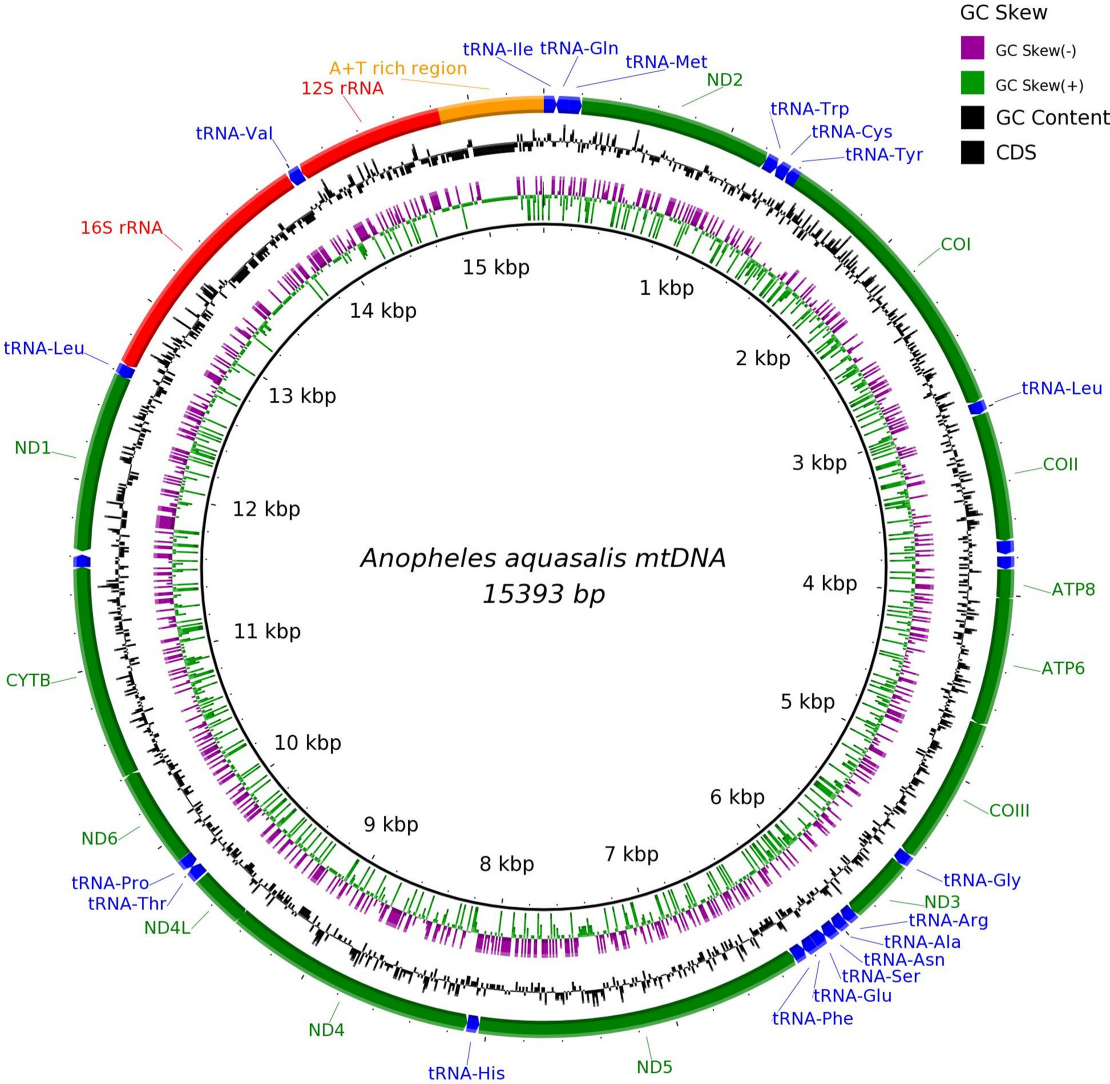
The complete mitogenome of *A*. *aquasalis*. BRIG visualization showing the protein coding genes, rRNAs and tRNAs in the mtDNA genome of *A*. *aquasalis*. The black inner ring shows the GC content on the outer surface, whereas AT content is shown on the inner surface. Strand asymmetry of the mitogenome is shown by the GC (+) and (-) skews according to the color key shown in the legend.

The complete mitogenome of *A*. *aquasalis* was assembled into a single contig of 15,393bp. The expected 37 genes in animal mtDNA, comprising 13 protein-coding genes, two rRNA genes (*12S* and *16S*), 22 tRNA genes and a control region were identified (shown in Figs 1 and 2). Aaquasalis_mt.gb, shows the annotated features in GenBank format. [NCBI as part of the *Anopheles aquasalis* whole genome shotgun sequencing project; accession number NJHH00000000]. A short region of 229 bp, located within the A-T rich region, failed to be assembled due to low coverage. That is consistent with the report showing lower coverage of the AT-rich region using Illumina Nextera kit than some other enzymatic sheering protocols [51].

The annotated genes are encoded on both the heavy (22 genes) and light (15 genes) strands with some ORFs overlapping adjacent genes. In total, there are 38 overlapping nucleotides between 14 neighboring genes with the junctions spanning from 1 to 7 bp. Excluding the control region, we found 44 intergenic nucleotides (IGNs) at 7 locations with their lengths ranging from 1 to 17 bp. As in other dipterans and metazoans [2, 25, 52], the most common start codon was ATG (6 PCGs). Incomplete or truncated termination codons were annotated in the following PCGs: *COI*, *COII*, *COIII*, *ND5*, and *ND4* (Fig 1). Other Anopheline mosquitoes also shared the same incomplete termination codons.

The mtDNA of *A*. *aquasalis*, as in other anophelines or insects [15, 53] includes 22 tRNA genes with anticodons representing 20 different amino acids, with a length ranging from 64–72 bp, and a total length of 1477 bp when concatenated. The lengths of the *12S* and *16S*rRNA genes are 793 and 1324 bp respectively, both being encoded on the light strand (L). As suggested for metazoans, the ends of both rRNA genes were assumed to stretch up to the boundaries of flanking genes [54]. Like reported for other dipterans species, the *16S*rRNA gene is flanked by the *tRNA*^*Leu*^ and *tRNA*^*Val*^ genes while the *12S*rRNA gene is placed between *tRNA*^*Val*^ and the control region. The A-T content for both was 82.5% and 79.9% respectively, resembling the composition of other dipterans as compared by Zhao *et al*., (2013). [25]. The 22 predicted secondary structures of individual tRNAs are shown in S1 Fig. All folded into the classic cloverleaf secondary structure except *tRNA-Ser* (GCT) in which the dihydrouridine stem “simply” formed a loop.

The nucleotide composition of the *A*. *aquasalis* mtDNA is biased towards a high A+T content. The overall AT% content in the whole mitochondrion sequence was 77.16%, and concordantly it had a 22.84% GC. The AT% for individual PCG's, long and short rRNA genes, and concatenated rRNAs and tRNAs are summarized in S1 Table.

Base composition, measured by strand asymmetry analyses (AT and GC skews), were also computed and are shown in S1 Table. Regarding strand compositional biases, whole anopheline mitogenomes have AT- and GC-skews that are similar to the reported for other metazoans [16, 55]. The complete *A*. *aquasalis* mitogenome has a positive AT skew and a negative GC skew for the majority strand (also known as light -L-), which means that this strand is richer in A and C nucleotides.

The *A*. *aquasalis* mitogenome contains 3743 codons whose usage is shown in S2 Table. Almost all the codons were present except AGG (S). There are 29 codons (out of 32) of the NNU and NNA types with a relative codon usage bias (RSCU) greater than 1. A strong bias towards A+T rich codons was observed, being the five most prevalent codons (in descending order): UUA (L), UUU (F), AUU (I), AUA (M), and AAU (N) as shown in S3 Table. The most used codon is UUA (L) and the less used codon in the genome is CGC (R). The mitogenome is rich in leucine while cysteine is the rarest amino acid.

### Comparative composition and identity analyses between selected anophelines

Compositional patterns based upon %AT and strand asymmetries were sought by comparing the mtDNA sequences from *A*. *aquasalis* and other human malaria vectors selected for their representative geographic distribution. The computed data and comparative approach rendered the plots shown in S2 Fig. Overall, the plots represent the similar trends of AT% and skew patterns between the compared features, amongst the evaluated anophelines. Matching profiles between invertebrates (mollusks) had been reported before by Plazzi *et al*., (2013) [52] via these types of plots. The observed trend had a few exceptions like the estimated GC skews from *COI* (from *A*. *darlingi* North and *A*. *darlingi* South) and *COII* (from *A*. *darlingi* South).

When comparing *A*. *aquasalis* coding nucleotide sequences and rRNA genes with their corresponding counterparts from the selected anopheline species, higher identity values were obtained with the American anophelines than with those from Africa and Asia. This was the general trend across all the compared PCGs and rRNA genes (S4 Table and S3 Fig).

### Phylogeny and molecular dating analyses

The PCG sequences of 18 *Anopheles* mtDNA genomes were analyzed including the following outgroups: *D*. *melanogaster*, *C*. *pipiens*, *Ae*. *albopictus*, and *Ae*. *aegypti* (GenBank numbers are shown in Table 1). The aligned and concatenated sequences from the 22 mitogenomes resulted in a block of 11,514 nucleotides. According to the Akaike Information Criterion, the best nucleotide substitution model for this data set was the General Time Reversible with gamma distribution (GTR + G) model.

**Fig 3:**
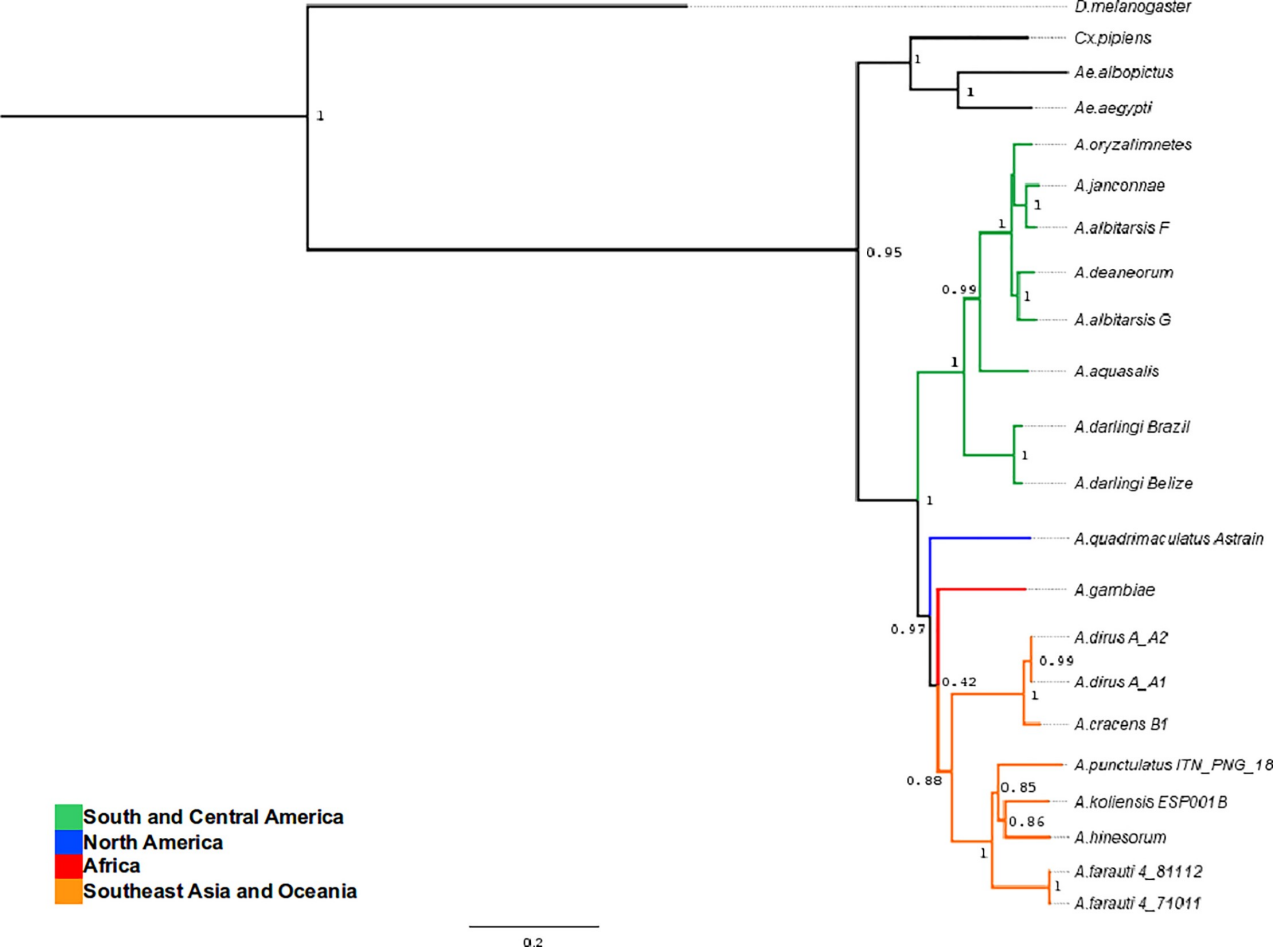
By-geographic region phylogeny of representative *Anopheles*, using the concatenated DNA sequences of all the mitochondrial protein coding genes. The values on the nodes correspond to the posterior probabilities supporting the tree topology. The phylogenetic tree was reconstructed using the concatenated PCGs and the Bayesian Markov Chain Monte Carlo approach (MCMC) analysis implemented in BEAST v1.7.5.

A phylogenetic tree was reconstructed using Bayesian analysis with BEAST v1.7.5 [49]. All the phylogenetic relationships were supported with robust posterior probabilities greater than 90%, with the exception of the position of *A*. *gambiae* (42%) and the internal nodes amongst the South East Asia and Oceania anophelines included (ranging from 85 to 88%). The reconstructed *Anopheles* phylogenetic tree is shown in Fig 3. From examining the tree topology, and considering the current continental distribution, a deep divergence between two *Anopheles* geographical lineages was observed. One clear monophyletic branch grouping Central and South American anophelines, and a second lineage grouping North American, Asian/Oceania, and African anophelines. As expected, this radiation pattern resembles the one published by Logue *et al*., (2013) [6]. The monophyletic clades corresponding to the *A*. *punctulatus*, *A*. *dirus* and *A*. *albitarsis* groups are also evident.

**Fig 4:**
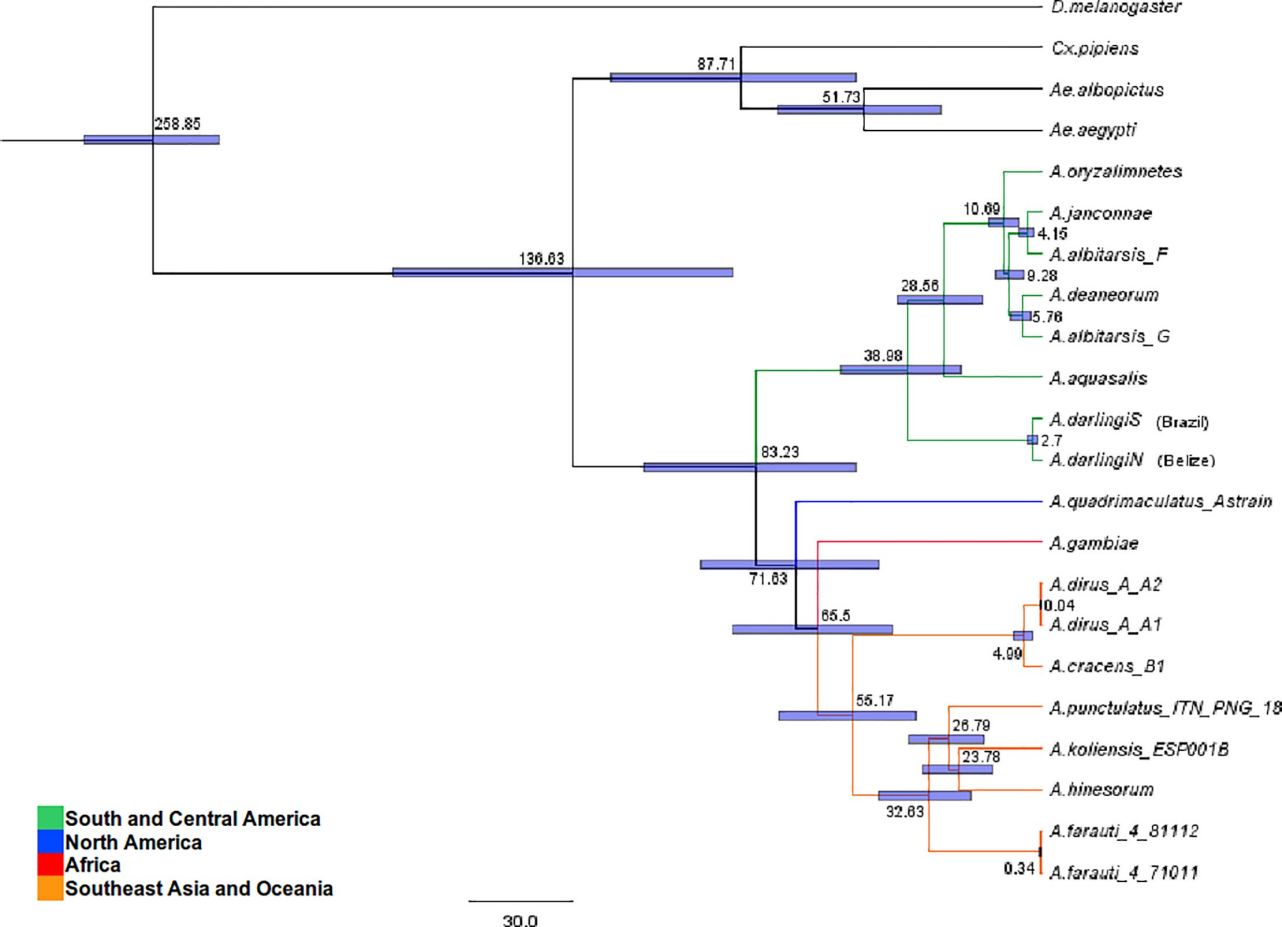
Phylogenetic tree of selected *Anopheles* using the concatenated DNA sequences of all the mitochondrial protein coding genes. The values on the tree nodes correspond to the mean divergence time (MYA) estimated for each event. The bars illustrate the 95% credibility intervals of the divergence times. (Table 2 presents the selected key divergence events).

The currently available fossil record for mosquitoes is poor [7, 56]. Thus, we estimated the divergence times amongst anopheline species using the *Drosophila*-*Anopheles* divergence time (set at 260 MYA) as the only calibration point time as previously suggested [6, 50]. We dated the most recent common ancestor (MRCA) of all *Anopheles* to 83.23 MYA with a 95% credibility interval ranging from 54.33 to 115.88 MYA as shown in Table 2. Amongst Neotropical anophelines, the MRCA within the *A*. *albitarsis* complex and *A*. *aquasalis* dates to 28.56 MYA with a 95% credibility interval ranging from 17.10 to 42.12 MYA. This MRCA is younger than the one shared between *A*. *darlingi* and the *A*. *albitarsis* complex, which dates back to 38.98 MYA as it can be observed in Table 2 and Fig 4.

**Table 2:** Mean divergence times in MYA (million of years ago), and 95% credibility intervals for selected nodes.

*Drosophila* / *Anopheles* (Calibration point ~260mya)	258.85	[239.70–278.86]
*Anophelinae* / *Culicinae*	136.63	[90.13–189.15]
*Anopheles* genus	83.23	[54.33–115.88]
*Anopheles gambiae* / SE Asia—Oceania anophelines	65.50	[43.54–89.80]
*Anopheles darlingi* / *Anopheles albitarsis* complex	38.98	[23.54–58.32]
*Anopheles aquasalis* / *Anopheles albitarsis* complex	28.56	[17.10–42.12]

Output of the analysis performed with BEAST v1.7.5 as explained in the Methods section. The measures in the second and third column correspond to the mean ages, and 95% credibility intervals determined for each of the selected nodes respectively.

## Discussion

Greater integration of nuclear and mitochondrial genomic studies is necessary to further our understanding of the *Anopheles* genomic evolution, the phylogenetic relationships between vectorial competence traits, and the co-evolutionary history of this genus with the human malaria parasites they may harbor. Thus, in the current “omics” era, extending the wealth of representative anopheline mitogenomes available is a necessary and feasible task.

Like in most metazoans, the assembled mitochondrial genome of *A*. *aquasalis* is a small, double-stranded, circular DNA molecule. Encompassed in 15,393 base pairs, we annotated a densely packaged set of 13 protein-coding genes, 22 tRNA genes, and two rRNA genes as shown in Fig 1. Genome structures with highly conserved features, composition, and organization have been reported among insects [51] such as anophelines [15].

When regarding genome architecture, the occurrence of overlapping open reading frames implies a genome with a compact structure. This feature can be observed by mtDNA sequences of culicines, anophelines, and other insect mitogenomes [47, 52, 53]. The reason for such characteristic can be (structurally) attributed to the small intergenic distances between consecutive genes (intergenic distance < x bp) as the following show: < 23 bp in *Ae*. *aegypti*; < 24 bp in *C*. *quinquefasciatus*; < 21 bp in *Ae*. *albopictus*; < 16 bp in *A*. *gambiae*; < 18 bp in *Anopheles quadrimaculatus*; < 30 bp in *Drosophila yakuba*, and < 30 bp in *D*. *melanogaster* (as recounted by [53]), and in the case of *A*. *aquasalis*< 17 bp. Once a free-living prokaryote, mitochondria underwent genome reduction as it transitioned into an obligate endosymbiont harboring tightly packed remnants from the eubacterial genome of its ancestor [54].

The intergenic distances do not account for the control region, which in the *A*. *aquasalis* mitogenome spans 558bp. This major non-coding region is also known as the A+T-rich region, and it plays a role in the initiation of transcription and replication [55]. The length of this region is highly variable among different insects due to the high rate of nucleotide substitutions, insertions/deletions, and a variable number of tandem repeats it can have [56, 57].

Almost all the tRNA sequences annotated could be folded into typical cloverleaf secondary structures (S1 Fig) exposing the adequate anticodon triplet, except for the DHU-arm of tRNA^Ser^, which is absent as it has been observed in other insects Li *et al*., 2013; Zhao *et al*., 2013 [25, 47] and references therein. Deficiencies regarding secondary structures of tRNA are often observed in protozoans, fungi, algae, plants and low metazoans [58]. The causes of such deficiencies range from aberrant loops and non-Watson-Crick matches to short arms, and all may induce aberrant tRNAs to lose their function, but a “corrective” post-transcriptional RNA editing mechanism has been proposed [59, 60]. The tRNA genes are embedded in variable regions within the mitogenome [51]. Throughout evolution, these regions underwent rearrangements more often than protein-coding regions. Consequently, tRNA order is nowadays explored as an additional tool for enhanced comparative phylogenetic analysis as between species [61].

The nucleotide composition of the complete *A*. *aquasalis* mtDNA sequence is clearly biased towards a high A+T content (77.16%) as it can be seen in S1 Table. This result was similar to the base composition described for other anophelines. The majority strand (L) of the *A*. *aquasalis* mtDNA has a compositional bias (positive AT skew and a negative GC skew). This means that this strand is richer in A and C. Though this is the tendency in insects, exceptions exist in arthropod mitogenomes in which the trend is reversed towards a composition with less A & C than T & G on the majority strand [62]. The underlying mechanism that leads to the strand bias, though unknown, has been linked to asymmetric replication and transcription processes. During both, one strand is transiently in a single-stranded state, and thus, it is left more prone to DNA damage. This phenomenon has been widely considered to account for the biased occurrence of mutations between the mtDNA strands [63].

Most of the start codons of the annotated PCGs followed the ATN rule, as described previously for other anophelines [15, 64]. Nonetheless, this was not the case for *COI* and *ND5*; genes that have TCG and GTG start respectively (Fig 1). The latter start codon characteristic has also been reported in other anopheline mitogenomes [15, 64, 65]. As observed in *A*. *darling* [15], eight genes use the complete stop codon TAA except for *COI*, *COII*, *COIII*, *ND5*, and *ND4*, which terminate with a single T (Fig 1). The incomplete stop codon is a reported phenomenon for insect mitogenomes [15, 25, 49, 66]. It has been proposed that the completion of the TAA termination codon is due to a post-transcriptional polyadenylation synthesis [67].

The total number of non-stop codons within the predicted PCGs (3743) was similar to that reported for *A*. *darlingi* and *A*. *gambiae* (3733), differing slightly to that of *A*. *quadrimaculatus* (3715). The nucleotide bias was also reflected in the codon usage within the annotated PCGs. A strong bias towards A+T rich codons like TTA (L), TTT (F), ATT (I), ATA (M), and AAT (N) is a compositional tendency that follows what has been reported as the canonical pattern for Culicidae species [15, 66]. When compared to other insects, such as aphids, the same five codons were the most prevalent, differing only in the exchanged F and I positions [49].

Among the 32 most frequently used codons (RSCU > 1) 29 were of the NNU and NNA type. This indicates that the third position of the codons mostly are either U or A. This differs from the expected trend for dipterans in which, frequently used codons present G or C in the third position [53].

The mitochondrial genome of insects displays unique characteristics such as high codon bias, low GC_3_content, and a highly conserved gene arrangement [51]. These characteristics are associated with the conserved patterns of Shine-Dalgarno sequences found in the transcripts of functional proteins. In addition, the aforementioned characteristics promote the high expression level of the mitochondrial genes [68, 69]. As a consequence of the above, mitochondrial activity of insects and nematodes would render them more tolerant to increased concentrations of reactive oxygen species (ROS) [70]. In the case of anophelines, it is interesting to consider linking this enhanced cellular resistance hypothesis with the detoxification mechanisms [71] towards the oxidative stress triggered as a response to the immune challenge posed by the *Plasmodium* infection [72, 73].

The sequence identity comparison and compositional patterns analyses we performed showed how the nucleotide sequence of protein-coding genes are a conserved feature of anopheline mitogenomes, in which most differences have been reported allocated within the control region [15, 74]. In terms of sequence identity (all values > 86%), a pattern was observed (S3 Fig). In general, the *A*. *aquasalis* PCGs are more similar to those of the other Neotropical anophelines tested, when compared to those from *A*. *gambiae* and *A*. *punctulatus* (African and Asian malaria vectors). Higher sequence similarity could be obeying the phylogeographic and divergence history of anophelines. Considering that, on average, the highest similarity values were obtained with *A*. *albitarsis*, another brackish-water Neotropical anopheline, from which *A*. *aquasalis* diverged ~28 MYA (Fig 4). The identity between rRNA sequences ranged from 93% to 98% (S4 Table). Similar identity values described in aphids prompted Wang *et al*., (2013) [49] to suggest that the predicted and potentially conserved rRNA secondary structures could improve phylogenetic analyses between insect species.

Human malaria vectors from different species complexes are not closely related. This implies that some of the competence traits potentially arose (or were lost) independently, multiple times within the different extant anopheline lineages. Therefore, the evolutionary history of genes associated with vector competence might have been driven by rapid processes. This would imply that such genes are not highly conserved sequences with a single ancestor as origin [75]. The publication of 16 anopheles genomes [8] might have generated, concomitantly, mitogenomes that will broaden our perspective on the phylogenetic and divergence relationships between the members of the *Anopheles* genus. This would be particularly important since both our study, and Logue *et al*., (2013) [6], encountered the paradoxical situation regarding the scarcity of mitogenomes from dominant malaria vectors pertaining to Africa (until recently) [76, 77] and the East Mediterranean regions, areas still burdened by human malaria [78]. For instance, at the time of our study, we opted to leave out the mitogenome of *Anopheles funestus* due the amount of gaps in the mtDNA PCGs sequences (GenBank accession number NC008070). It was not until recently that mitochondrial lineages of this important vector were thoroughly studied [77].

The reconstructed *Anopheles* phylogeny showed a deep divergence between two main *Anopheles* lineages (Fig 3) seemingly driven by their phylogeographic relations and the earth's geologic eras. The tree topology obtained is consistent with the current hypothesis regarding the origin of *Anopheles* mosquitoes in the Gondwana supercontinent during the Cretaceous period [12, 14]. *Anopheles* mosquitoes would have radiated from what is recognized as South America, then arrived in Africa and from there then colonized Europe and North America (with the aid of land bridges), also migrating through Asia, and into the Pacific [6]

However, as previously mentioned, paucity regarding mitogenomes from African, Asian and European anophelines, hindered the chance of determining accurately if African anophelines are the ancestors of those in Europe and North America, or if North American anophelines radiated from South America. For instance, both our results and those of Logue *et al*., (2013) [6], represent a phylogeny in which the position of *Anopheles quadrimaculatus* seems unresolved.

Nonetheless, Freitas *et al*., (2015) [7] provides a thorough revision of phylogenetic relations based on COI, COII and 5.8S rRNA genes which allowed for more species to be tested. When comparing with it, we observed how the mitogenome based relations here reported are the same at the subgenus level as Neafsey *et al*., (2015) [8] and Krzywinski *et al*., (2001) [10], being (*Nyssorhynchus*, (*Anopheles*, *Cellia*)). Whereas their study was not able to significantly resolve the evolutionary relationship within this taxonomic level, other studies have reported different associations too, namely, *Cellia*, (*Nyssorhynchus*, *Anopheles*) [79]. A recent comparative evolutionary mitochondriomic study strongly supported the sister relationship of *Nyssorhynchus* + *Kerteszia* and *Cellia* + *Anopheles* based on 50 complete mitogenomes [80]. Thus, this evolutionary relationship seems to be conclusively elucidated at this taxonomic level.

It has been stated that interpreting the current distributions of anophelines from an evolutionary context, may be quite problematic [7]. This author reviewed existing alternative hypotheses, some suggesting a different scenario for the evolution of the extant groups of the *Anophelinae* subfamily. The scenario would reflect the ideas presented by Christophers (1933) [81], in which the ancestral lineage of *Anopheles* existed prior to the split of Pangaea, diversifying into the modern species after the breakup of the continents. Nonetheless, it is not the scope of this study to dig deeper into this matter.

Regarding the estimated divergence times, we dated the *Anopheles* MRCA to ~ 83 MYA. The node age (Fig 4) differs from the ~ 79 MYA reported by Moreno *et al*., (2010) [16], the ~ 93 MYA estimated by Logue *et al*., (2013) [6] and 110 MYA by Freitas *et al*., (2015) [78]. These molecular dating estimates, though different, are in a degree of agreement with the breakup of Western Gondwana and the loss of land connections between South America and Africa (90–95 MYA). This geologic event might have prompted the divergence between both *Anopheles* + *Cellia* from *Nyssorhynchus*, and further on, the divergence between *Cellia* and *Anopheles* ~ 71 MYA or ~ 81 MYA [6]. The geographic sorting of lineages thus would coincide with the loss of land bridges connecting Africa and Europe, as well as between Europe and North America. This, in turn, could account for the absence of *Cellia* in the New World and *Nyssorhynchus* in the Afro-Eurasian continents [10, 15]. In agreement with the above Freitas *et al*., (2015) [78] determined, by ancestral area reconstruction, that a monophyletic clade composed of the Neotropical subgenera Stethomyia, *Kerteszia* and *Nyssorhynchus* had a common ancestor distributed along the Americas, and whose early radiation in the continent began around the Late Cretaceous (~ 90 MYA).

The data suggest that *A*. *aquasalis* diverged from the *A*. *albitarsis* complex ~ 28 MYA. The node age implies that their MRCA would be younger than the one shared between *A*. *darlingi* and the *A*. *albitarsis* complex which dates back to ~ 38 MYA (Fig 4). If Neotropical anophelines bionomics are considered, then *A*. *aquasalis* would have diverged from the *A*. *albitarsis* complex, adapting to its narrow coastal ecological niche, outcompeted by inland (*A*. *darlingi*) and brackish-water adapted (*A*. *albitarsis*) sibling species [82].

As brought forward by Freitas *et al*., (2015) [78], when comparing their results to Fontaine *et al*., (2015) [9], phylogenetic reconstructions for *Anopheles* require robust data from multiple molecular markers, and individuals per species. It is highlighted by the authors that unclear phylogenies regarding African anophelines, are due to complex speciation processes that have been permeated by recurring events of introgressive hybridization. Nonetheless, it drew our attention that, based on X chromosome phylogenies (which reflect species branching order), Fontaine *et al*., (2015) [9], determined that brackish water adapted *Anopheles merus* and *Anopheles melas* [83], diverged from bionomical different inland sibling species ~1.85–1.47 MYA, whereas *A*. *aquasalis* might have diverged from inland sibling *A*. *darlingi* ~38.98 MYA. As evidence for phylosymbiosis emerges, including examples of non-brackish water adapted or Neotropical anophelines [84], it becomes relevant to assess if co-evolution of *A*. *aquasalis* and its aquatic niche acquired microbiota, follow this trend. As a growing field of interest [85–87], exploring the ecological adaptations of anophelines to particular breeding sites, and its connection to their microbiota signature profiles, should generate interesting insights when comparing similar bionomical species for example.

The observed differences regarding radiation and divergence node ages have been reported by other authors [7, 12, 15]. Notably, the choices regarding data inclusion have a direct effect on phylogenomic studies. Therefore, the use of full mitogenomes, individual or concatenated genes (PCGs, rRNAs, tRNAs) or nuclear genes, can be accounted as the reason for the differences between molecular evolutionary histories reported to date [16, 51]. Additionally, observed discrepancies may also be due to: depth of species sampling while seeking for representatives of the geographical regions and /or the subgenera studied; the choice (and use) of mutation rates; and the amount of calibration points employed [6, 12, 15]. For example, Moreno *et al*., (2010) [15] used as an additional calibration point, the Anophelinae and Culicinae divergence age estimated to be 120 MYA [88]. Nevertheless, the most rigorously calculated date available involving the split between a mosquito lineage and a sister taxon is the divergence time of 259.9 MYA between *Drosophila* and *Anopheles* estimated by Gaunt & Miles, (2002), as highlighted by Krzywinski *et al*., (2006) [12, 44]. As there are many pitfalls inherent to the use of molecular clocks to date divergences, the outcome of age estimation should be interpreted with caution [12].

Molecular dating of the divergence events reported in this study occurred long before humans arrived in America 15–20 millennia ago [89]. Evidence suggests that *Plasmodium* parasites were introduced into the Americas via African slave trade routes during the European invasion [20]. In order to the transmission cycle to establish, the parasite had to adapt to the populations of local mosquitoes. Then, in accordance with the present geographic distribution of anophelines [17], and the known slave trade disembarking ports, some of the first indigenous vectors that *P*. *falciparum*-infected humans encountered would have been: *A*. *albimanus*, *A*. *aquasalis*, *A*. *darlingi*, *A*. *albitarsis* and *A*. *quadrimaculatus* [90].

## Conclusion

Considering the evolutionary distance between Old and New World anophelines, it is not surprising that there are marked differences at genetic, ecological and behavioral levels between them [17]. This probably resulted in a more stringent selection upon *Plasmodium* as it adapted to these new vectors [90]. Multiple Neotropical anophelines became human malaria vectors independently from each other, including the anophelines from South East Asian and Oceania [6]. This suggests that co-occurrences of anopheline traits related to malaria transmission would be the outcome of convergent evolution [91].

Readily available anopheline phylogenies are key to perform co-evolutionary studies between parasites and vectors to gain insights into their interspecific relationships [7]. Recently, Molina-Cruz & Barillas-Mury, (2014) [90], proposed a hypothesis linking the *Plasmodium* protein PFS47, and a still unknown interacting protein from *Anopheles*, acting as a critical determinant of mosquito-parasite compatibility and thus the “emergence” of vector competence in this genus [90, 92, 93]. Considering that the *Pfs47* gene is polymorphic, and presents a robust clonal distribution, we support the need of a better and more accurate *Anopheles* phylogeny. This, in turn, could enable the reconstruction of the history of malaria transmission in the New World.

## Supporting information

S1 Fig *A. aquasalis* mtDNA predicted tRNA structures. 22 tRNAs were identified in the mitogenome of *A*. *aquasalis* and their cloverleaf secondary structures predicted with RNAStructure. Using Dayhoff’s single letter amino acid code.
(TIF)

S2 Fig Compositional patterns of *A. aquasalis* and other anopheline mitochondrial genomes. A-T content expressed as AT%, AT skew, and GC skew were estimated and plotted for each single PCG and for other genomic regions according to the legend below the chart. Refer to the S5 and S6 Tables to access the tabular data regarding each parameter.
(TIF)

S3 Fig Radar depiction of the nucleotide identity comparison between mitochondrial PCGs and rRNA genes from *A. aquasalis* and other anophelines. Nucleotide identity (%) between *A*. *aquasalis* and selected *Anopheles* species ranging from 80 to 100%. The scale is shown along the main vertical axis of the radar plot.
(TIF)

S1 Table Base composition analysis of the *A. aquasalis* whole mitochondrion DNA sequence, and other annotated features such as individual PCGs, and concatenated rRNA & tRNA genes. (XLSX)

S2 Table Codon usage in the *A. aquasalis* mtDNA. Codon frequency and Relative synonymous Codon Usage (RSCU) are shown for each codon.
(XLSX)

S3 Table Top 10 codons (classified by count) identified in the protein coding genes of the *A. aquasalis* mitogenome. (XLSX)

S4 Table Protein coding genes nucleotide sequence identity comparison between *A. aquasalis* and other selected anophelines. (XLSX)

S5 Table Comparative strand asymmetry analysis based on AT and GC skews between the *A. aquasalis* mtDNA and selected anopheline species. (XLSX)

S6 Table Comparative compositional analysis represented in AT% between the *A. aquasalis* mtDNA and selected anopheline species. (XLSX)
