# Supplementary figures and images for: Characterization of the complete mitogenome of Anopheles aquasalis, and phylogenetic divergences among Anopheles from diverse geographic zones
Source: PLoS One. 2019 Sep 3;14(9):e0219523. doi: 10.1371/journal.pone.0219523 (PMC6720026; doi:10.1371/journal.pone.0219523)

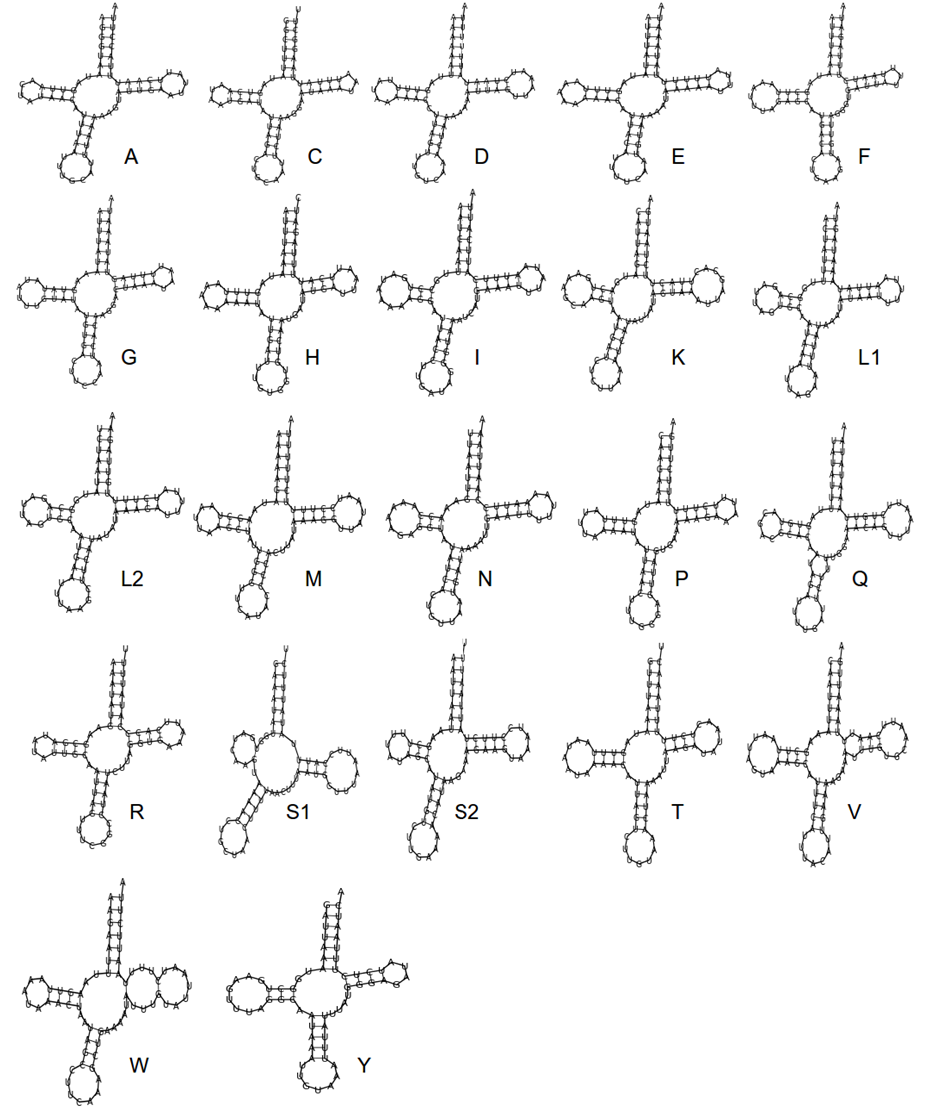

Supplement: S1 Fig — 22 tRNAs were identified in the mitogenome of A. aquasalis and their cloverleaf secondary structures predicted with RNAStructure. Using Dayhoff’s single letter amino acid code. (TIF) [file pone.0219523.s001.tif]

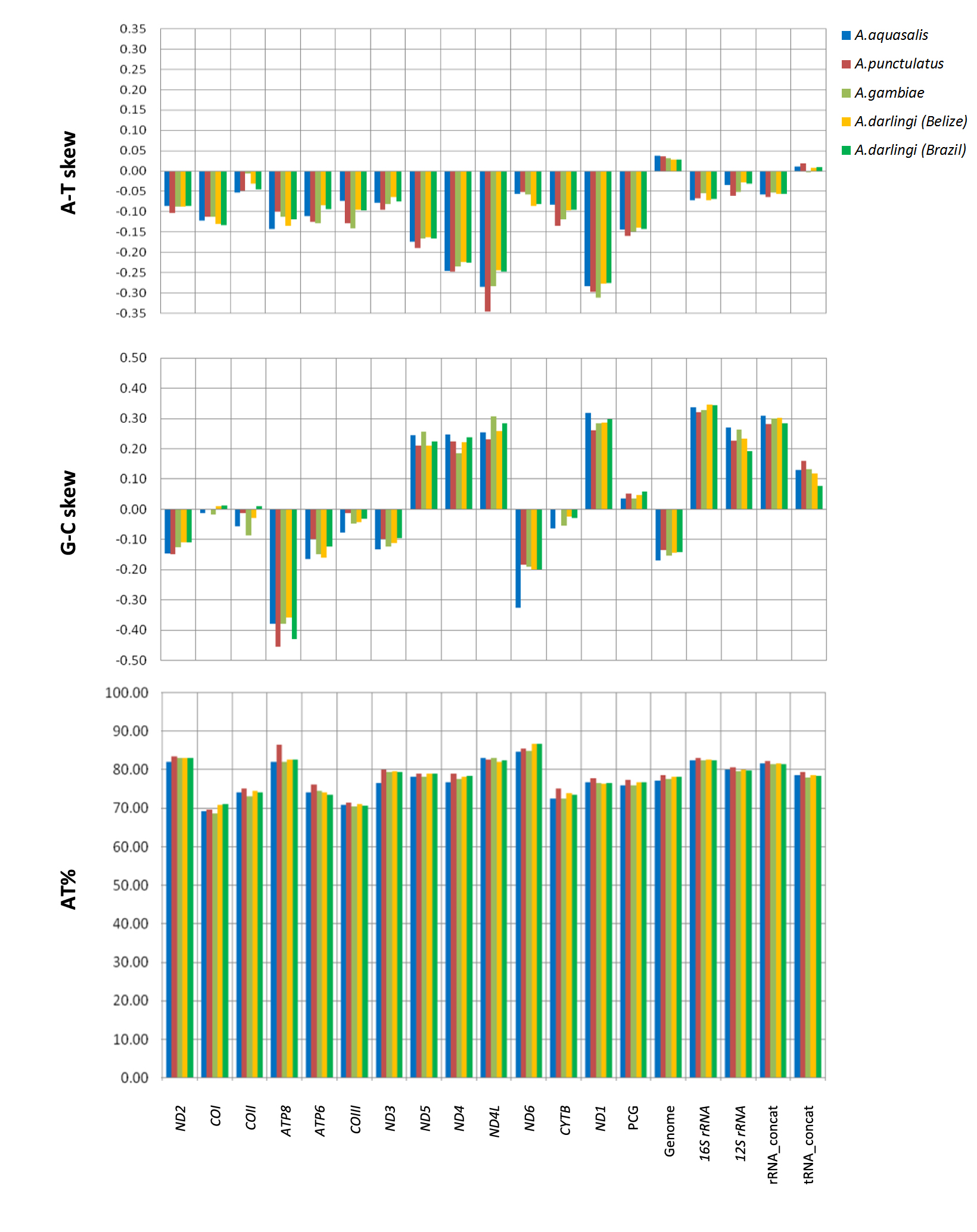

Supplement: S2 Fig — A-T content expressed as AT%, AT skew, and GC skew were estimated and plotted for each single PCG and for other genomic regions according to the legend below the chart. Refer to the S5 and S6 Tables to access the tabular data regarding each parameter. (TIF) [file pone.0219523.s002.tif]

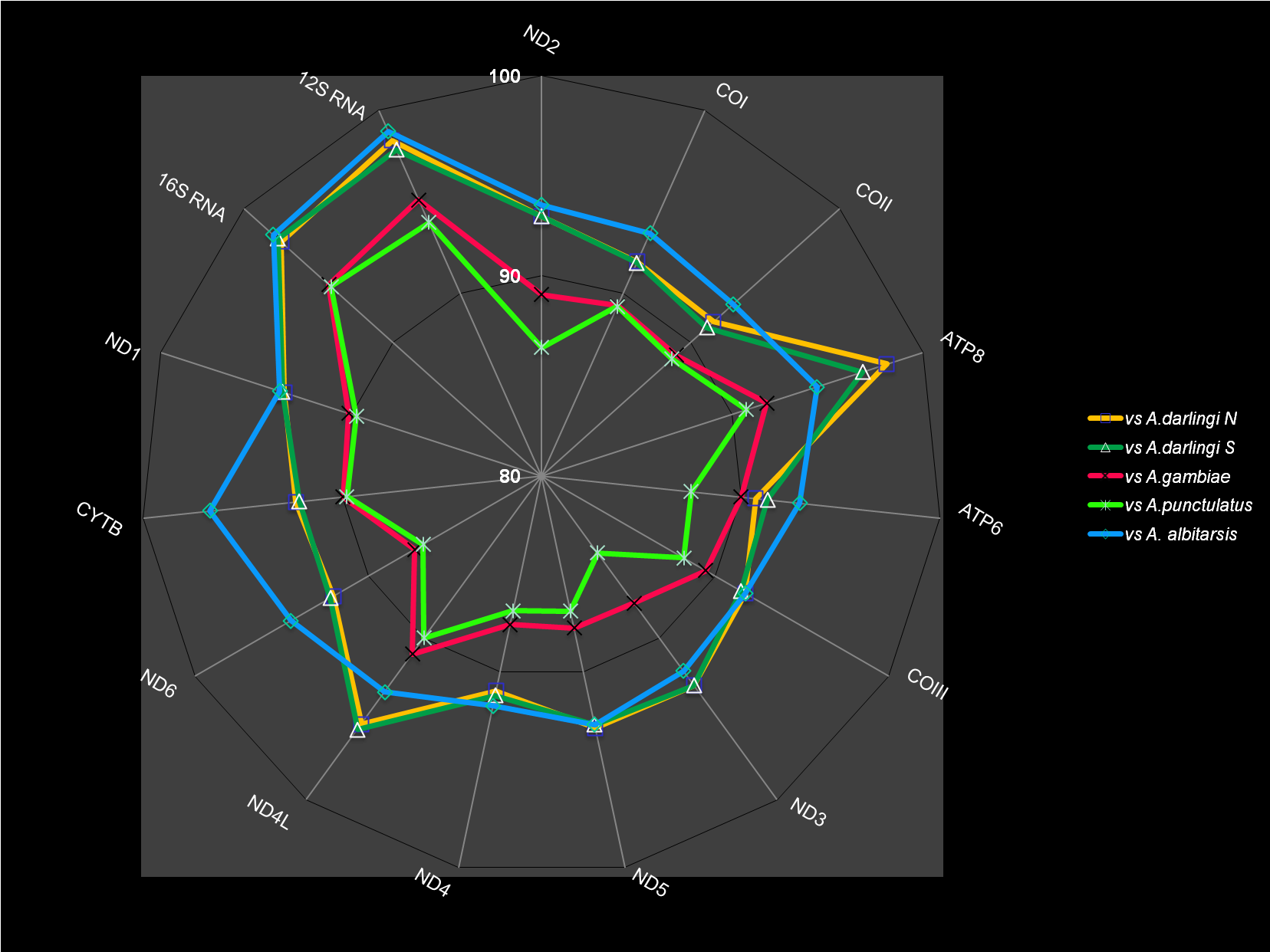

Supplement: S3 Fig — Nucleotide identity (%) between A. aquasalis and selected Anopheles species ranging from 80 to 100%. The scale is shown along the main vertical axis of the radar plot. (TIF) [file pone.0219523.s003.tif]
